# Anatomic, histologic, and mechanical features of the right atrium: implications for leadless atrial pacemaker implantation

**DOI:** 10.1093/europace/euad235

**Published:** 2023-07-31

**Authors:** Matthew O’Connor, Umberto Barbero, Daniel B Kramer, Angela Lee, Alina Hua, Tevfik Ismail, Karen P McCarthy, Steven Niederer, Christopher A Rinaldi, Vias Markides, John-Ross D Clarke, Sonya Babu-Narayan, Siew Yen Ho, Tom Wong

**Affiliations:** Department of Electrophysiology, Royal Brompton and Harefield Hospitals, Guy's and St Thomas’ NHS Foundation Trust, London SW3 6NP, UK; Department of Electrophysiology, Auckland City Hospital, Auckland, New Zealand; Cardiac Morphology Unit, Royal Brompton Hospital, London, UK; Cardiology Unit, Santissima Annunziata Hospital, Savigliano ASL CN1, Italy; Richard A. and Susan F. Smith Center for Outcomes Research, Beth Israel Deaconess Medical Center, Harvard Medical School, Boston, MA, USA; School of Biomedical Engineering and Imaging Sciences, King’s College London, London, UK; School of Biomedical Engineering and Imaging Sciences, King’s College London, London, UK; Department of Cardiology, Guy’s and St Thomas’ Hospital Guy's and St Thomas’ NHS Foundation Trust, London, UK; Department of Cardiology, Kings College Hospital, London SE5 9RS, UK; Cardiology Unit, Santissima Annunziata Hospital, Savigliano ASL CN1, Italy; School of Biomedical Engineering and Imaging Sciences, King’s College London, London, UK; Department of Cardiology, Kings College Hospital, London SE5 9RS, UK; Department of Electrophysiology, Royal Brompton and Harefield Hospitals, Guy's and St Thomas’ NHS Foundation Trust, London SW3 6NP, UK; Division of Cardiovascular Medicine, Beth Israel Deaconess Medical Center, Harvard Medical School, Boston, MA, USA; Department of Electrophysiology, Royal Brompton and Harefield Hospitals, Guy's and St Thomas’ NHS Foundation Trust, London SW3 6NP, UK; Cardiac Morphology Unit, Royal Brompton Hospital, London, UK; Department of Electrophysiology, Royal Brompton and Harefield Hospitals, Guy's and St Thomas’ NHS Foundation Trust, London SW3 6NP, UK; Department of Cardiology, Kings College Hospital, London SE5 9RS, UK; Department of Cardiology, National Heart & Lung Institute, Imperial College London, London SW3 6LY, UK

**Keywords:** Leadless pacing, Atrial anatomy, Right atrial appendage, Dual chamber pacing, Aveir pacemaker

## Abstract

**Background:**

Leadless pacemakers (LPs) may mitigate the risk of lead failure and pocket infection related to conventional transvenous pacemakers. Atrial LPs are currently being investigated. However, the optimal and safest implant site is not known.

**Objectives:**

We aimed to evaluate the right atrial (RA) anatomy and the adjacent structures using complementary analytic models [gross anatomy, cardiac magnetic resonance imaging (MRI), and computer simulation], to identify the optimal safest location to implant an atrial LP human.

**Methods and results:**

Wall thickness and anatomic relationships of the RA were studied in 45 formalin-preserved human hearts. *In vivo* RA anatomy was assessed in 100 cardiac MRI scans. Finally, 3D collision modelling was undertaken assessing for mechanical device interaction. Three potential locations for an atrial LP were identified; the right atrial appendage (RAA) base, apex, and RA lateral wall. The RAA base had a wall thickness of 2.7 ± 1.6 mm, with a low incidence of collision in virtual implants. The anteromedial recess of the RAA apex had a wall thickness of only 1.3 ± 0.4 mm and minimal interaction in the collision modelling. The RA lateral wall thickness was 2.6 ± 0.9 mm but is in close proximity to the phrenic nerve and sinoatrial artery.

**Conclusions:**

Based on anatomical review and 3D modelling, the best compromise for an atrial LP implantation may be the RAA base (low incidence of collision, relatively thick myocardial tissue, and without proximity to relevant epicardial structures); the anteromedial recess of the RAA apex and lateral wall are alternate sites. The mid-RAA, RA/superior vena cava junction, and septum appear to be sub-optimal fixation locations.

## Graphical Abstract

**Graphical Abstract:**
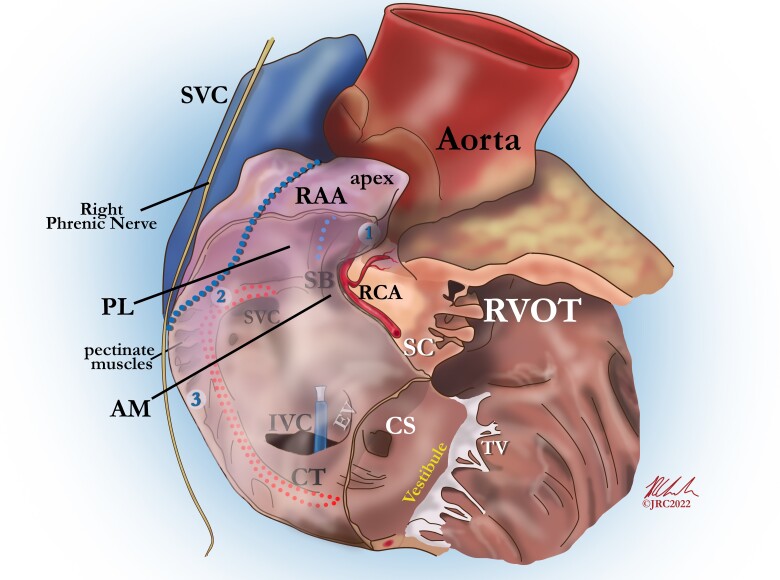
Attitudinal view of the right atrium demonstrating the three proposed optimal locations for the implantation of an atrial leadless pacemaker; (1) anteromedial RAA apex, (2) base of the RAA, (3) right atrial lateral wall. AM, anteromedial (aspect of the RAA); CS, coronary sinus; CT, crista terminalis; EV, eustachian valve; IVC, inferior vena cava; PL, posterolateral (aspect of the RAA); RAA, right atrial appendage; RCA, right coronary artery; RVOT, right ventricular outflow tract; SB, septal bundle; SC, supraventricular crest; SVC, superior vena cava; TV, tricuspid valve.

**What’s new?**

- Leadless pacing in the atrium has unique anatomical considerations.
- The optimal implant location for an atrial leadless pacemaker is as yet undefined.
- From an anatomical and functional perspective the best compromise for an atrial LP implantation may be the RAA base.
- The anteromedial recess of the RAA apex and lateral wall are alternate sites.
- The mid-RAA, RA/SVC junction and septum appear to be sub-optimal fixation locations.

## Introduction

Traditional transvenous pacing systems are reliable but require patent superior vascular access and include cumulative risks such as lead failure, infection, pocket complications, and venous obstruction.^1–3^ Leadless pacemakers (LPs), implanted via the femoral vein, overcome many of these concerns and minimize the risk of device-related infection.^4^ Currently available LP systems provide limited atrioventricular (AV) synchrony for patients with sinus rhythm and cannot provide atrial pacing for isolated sinus node dysfunction.

An atrial LP in development (the Aveir^TM^ dual chamber leadless pacemaker system, Abbott Medical, Sylmar, CA, USA), is 32 mm long, 6.5 mm in diameter, and includes a non-retractable helix with an active fixation depth of approximately 1.63 mm. This device, currently under evaluation in the single-arm Aveir DR i2i Study (ClinicalTrials.gov Identifier: NCT05252702), may fill an important clinical niche but raises unique considerations for safe and effective implantation in the right atrium (RA). The increased cost and complexity of a dual leadless pacemaker system need to be offset by the potential clinical advantages (optimal AV synchrony) that such a system may afford patients beyond the current LPs already in clinical use. Compared with right ventricular (RV) LP placement, RA implantation confronts a thinner myocardial wall, smaller chamber size, variable septal integrity, and complex right atrial appendage (RAA) geometry. Moreover, safe implantation must consider interaction with the phrenic nerve, right coronary artery, and great vessels, while also anticipating potential repetitive movement and intra-atrial mechanical irritation of the LP within the RA in concert with cardiac motion even after fixation. Clinical studies of ventricular LPs demonstrated the importance of implant technique and a procedural learning curve for safe and effective device deployment.^5,6^ Ideally, anatomic considerations and simulated implantation modelling would guide operator training and refine operative technique before widespread clinical deployment.

Motivated by these concerns, this study evaluated, in detail, the RA anatomy relevant to the implantation of an atrial LP. We leveraged a combination of detailed anatomic evaluation of human heart specimens, magnetic resonance imaging (MRI) of *in vivo* hearts, and computer-simulated implantation to model optimal implantation location for atrial LP. This report outlines findings from: (i) our anatomic evaluation focused on RA wall thickness and proximity to other structures relevant to implantation of an LP; (ii) MRI analysis of RA dimensions with regards to a 32.2 mm LP implant, a factor that has not been relevant in the implantation of transvenous leads in the RA; and (iii) computer modelling of a virtual implant evaluating dynamic interaction of an implanted/mobile LP in the RA throughout the cardiac cycle.

## Methods

All patients provided written informed consent and the study received institutional ethics approval. The study was conducted according to the declaration of Helsinki. The original data are available upon reasonable request to the corresponding author.

### Anatomical study of wall thickness, right atrial, and right atrial appendage dimensions

**Figure 1:**
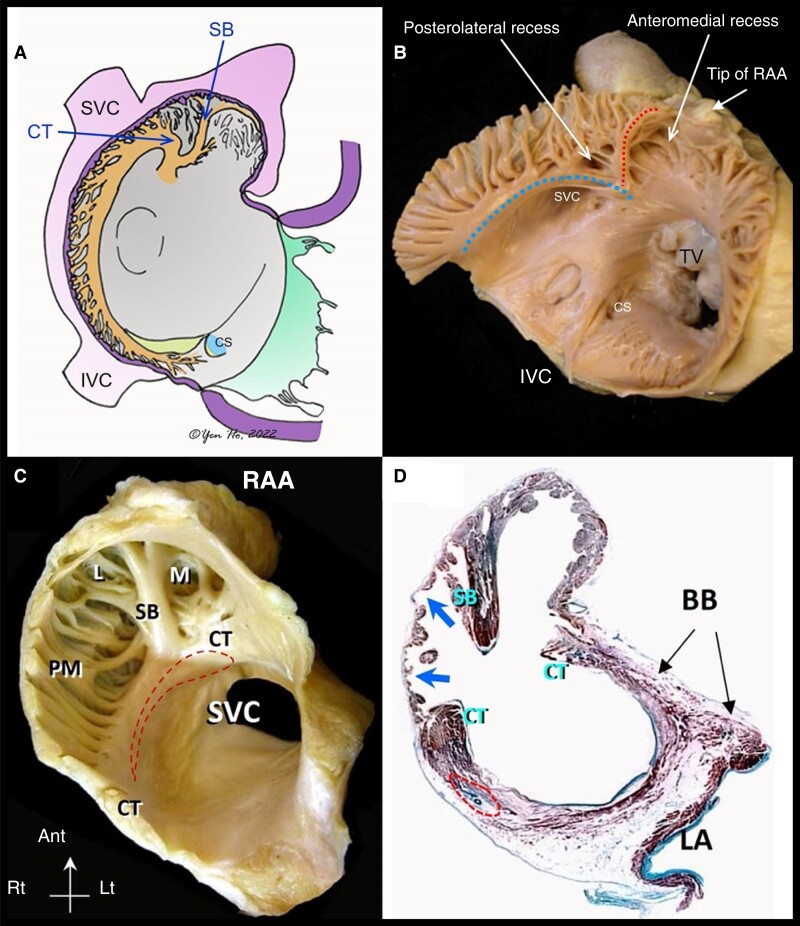
Endocardial right atrial anatomical relations: (*A*) diagram of right atrial appendage from an right anterior oblique (RAO) perspective. (*B*) Representative specimen of the RA demonstrating the CT [large (blue) dots] and its bifurcation with the SB [small (red) dots]. (*C*) Representative specimen of the RAA viewed in left anterior oblique (LAO). (*D*) Histological section through the RAA showing the thin wall (arrows) between pectinate muscles. BB, Bachmann’s bundle; CT, crista terminalis; L, posterolateral recess; LA, left atrium; M, anteromedial recess; PM, pectinate muscles; RAA, right atrial appendage; SB, sagittal bundle, SVC, superior vena cava. (*D* and *C*) reproduced with permission.^8^ CS, coronary sinus; CT, crista terminalis; IVC, inferior vena cava; RAA, right atrial appendage; SB, sagittal bundle; SVC, superior vena cava; TV, tricuspid valve.

We refined our gross anatomical evaluation based on practical clinical considerations. First, an atrial LP implant site in the septum was deemed inappropriate as the mobility of the septum would compromise device stability, while the presence of a patent foramen ovale in ∼20% of the population pertains an unacceptable risk of inadvertent LA placement of a device.^7^ Similarly, the vestibule was deemed inappropriate as a delivery system from a femoral approach would not be able to conform to the acute angle required for such an implant. Thus, potential implant locations identified for an atrial LP included; the RAA base [at the point of the superior crista terminalis (CT) sagittal bundle (SB) bifurcation], RAA body, RAA apex, and the RA lateral wall. We thus focused our study on these areas to further examine the anatomy and potential issues with each location (*Figure 1*).

**Figure 2:**
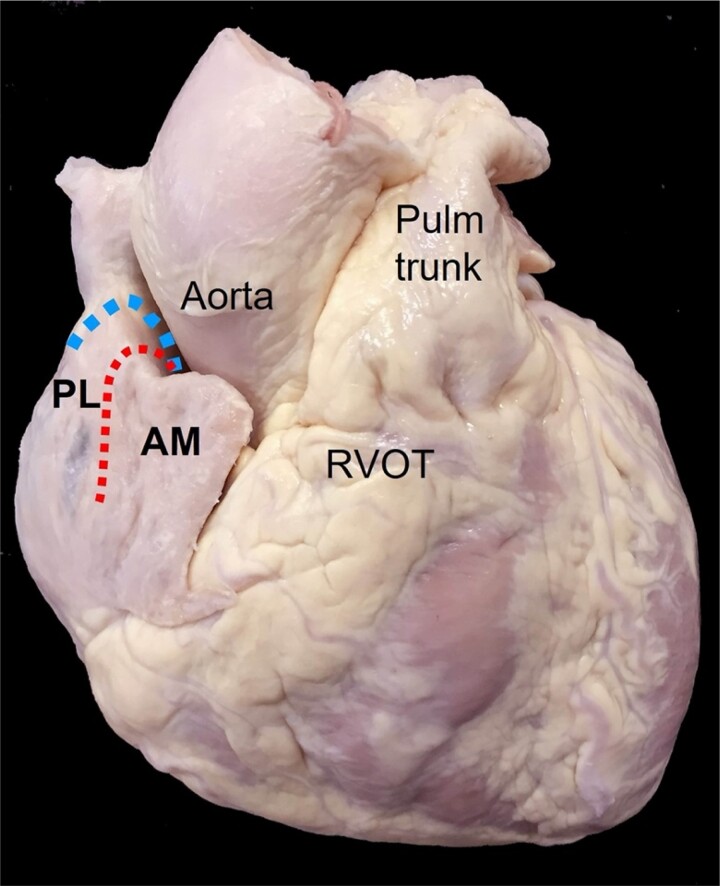
Epicardial right atrial anatomical relationships: anterior view of the heart demonstrating the right atrial appendage (RAA) and its relationship to the aorta and right ventricular outflow tract (RVOT). The RAA is divided into a posterolateral (PL) and anteromedial (AM) recess by the sagittal bundle [samll (red) dots] and the crista terminalis [large (blue) dots]. The epicardial fat pad overlying the RVOT and RCA can be seen just below the AM. RCA, right coronary artery.

We defined the RAA as the entire anterior, lateral, and supero-medial part of the RA, which is demarcated on the endocardial surface by the CT, delineating it from the posteriorly situated smooth-walled venous portion and the smooth-walled vestibule around the tricuspid valve orifice. For the purposes of the anatomical analysis, we divided the RAA apex into two regions (*Figure 2*), as originally described by McAlpine; the anteromedial and posterolateral recesses, divided by the SB.^9^

Right atrial wall thickness was evaluated by examination of 45 human hearts specimens that had been fixed by immersion in 10% formalin solution from the archives of the Cardiac Morphology Unit, Royal Brompton Hospital. Hearts with macroscopically visible structural abnormalities such as valvular stenosis or ventricular hypertrophy were excluded although we included hearts with minimal myocardial scarring.

Gross examination was performed to define and categorize the features and wall thickness of both the anteromedial and posterolateral recesses of the RAA apex and the remainder of the RAA and RA. For each location, width and transmural thickness from the endocardium to the epicardium were measured using callipers with the use of magnification through a Nikon SMZ-2T Stereo Microscope with halogen cold light source (Schott, KL 1500). Additionally, examination of histological sections of the RAA wall from five hearts was performed.

### Magnetic resonance imaging analysis of right atrial appendage dimensions

One hundred patients undergoing routine cardiac MRI (1.5 T scanners, Siemens Magnetom Avanto and Siemens Magnetom Aera) were prospectively enrolled. Specific inclusion and exclusion criteria are available in the Appendix.

During standard protocols, each patient was scanned with steady state free procession pulse sequences in order to obtain end-expiratory breath-hold cine images of the four-chamber long-axis view and a two-chamber long-axis view of the right ventricle. Right atrial and RAA dimensional analyses were performed with semi-automated software [Cardiac Magnetic Resonance imaging (CMR) tools, Cardiovascular Imaging Solutions, London, UK].

For each patient, we measured the dimensions of the RAA ostium and depth, the distance from the CT to relevant other anatomical structures, and the proximity of the right pulmonary pleura to the lateral RA wall. These measurements were chosen as they have important repercussions for an atrial LP placed in the RAA, CT, or lateral RA. All measurements were derived from 2D images were measured corresponding to the cine phase at which the atrial size was at its maximum.

### Virtual implantation of an atrial leadless pacemaker

3D dynamic modelling was undertaken of a virtual implantation of an atrial LP based on the Aveir^TM^ (Abbott Medical, Sylmar, CA, USA) leadless pacemaker (32.2 mm long, diameter 6.5 mm and volume 96 mm3) to evaluate for potential mechanical interactions of the atrial LP within the RA. Technical methodology was adapted from our previous work in modelling an LP’s motion within the RV cavity.^10^ Cine CMR 2D atrial stack images were acquired from 10 healthy individuals (see Supplementary material online, *Table S3*). Right atrial motion was tracked using feature tracking within Cardiac Electro-Mechanics Research Group Application (CemrgApp; cemrgapp.com).^11^ Manual RA cavity segmentation was performed, an RA endocardial triangulated mesh was generated and the image registration warping field was applied to mesh. All 10 patient models had a virtual atrial LP implanted at each vertex of the triangulated endocardial mesh orientated normal to the mesh surface. The LP was then moved with warped mesh during the cardiac cycle and contact with the rest of the mesh for each LP location was scored as a proportion of the cardiac cycle. This results in each location being assigned a contact value as a percentage of the cardiac cycle. We modelled the tricuspid annulus as an additional atrial wall and considered collision with this wall to be clinically significant as it represents interaction with the tricuspid valve.

## Results

### Anatomical study of wall thickness, right atrial, and right atrial appendage dimensions

#### Crista terminalis/septal bundle bifurcation and right atrial appendage base

The CT itself was fundamentally a C-shaped structure which traversed from the anteromedial wall of the right atrium to the left of the superior vena cava (SVC). At its most superior position at the bifurcation with the SB, the CT had a mean width of 6.0 ± 1.5 mm (range 4–10 mm) and thickness of 4.9 ± 1.9 mm (range 2.5–9 mm) (*Figure 1*). This location corresponds to the septal aspect of the RAA base which is in close association with posterolateral recess and abuts the aortic root. Beyond the SB bifurcation, the CT continued laterally/inferiorly and gave rise to a series of pectinate muscles, which fanned out anteriorly towards the smooth vestibule of the tricuspid valve and into the RAA itself. In this region, the lateral aspect of the RAA base, ∼10 mm from the origin of the SB, the mean width of the CT was 5.6 ± 1.4 mm (range 3–9 mm) and mean thickness 4.2 ± 1.4 mm (range 2.5–8 mm) (*Table 1*).

**Table 1:** Wall thickness of potential RA LP implant locations

Location	Wall thickness (mm)
RAA base (10 mm from the origin of the CT)	4.2 ± 1.4
RA lateral wall	2.6 ± 0.9
Anteromedial RAA apex (anterolateral segment)	1.0 ± 0.5
Anteromedial RAA apex (lateral segment)	0.8 ± 0.4
Anteromedial RAA apex (posterior segment)	0.9 ± 0.6

CT, crista terminalis; LP, leadless pacemaker; RA, right atrium; RAA, right atrial appendage.

### Right atrial appendage body and apex

Gross examination of the endocardial surface of the RAA revealed a single SB bifurcating from the CT in 61%, multiple sagittal bundles in 27%, and absence of SB in 12% of the hearts. Thus, in 88% of cases, the SB clearly demarcated the RAA apex into two segments: a posterolateral recess, lying adjacent to the aortic root and an anteromedial recess, near, but not abutting the RV outflow/pulmonary artery.

Regarding the body of the RAA where the inner surface was dominated by pectinate muscles, the thickness of the pectinate muscles at three points (corresponding to anterolateral, lateral, and posterior segments of the tricuspid circumference) were 1.3 ± 0.4 mm, 1.2 ± 0.3 mm, and 1.3 ± 0.4 mm, respectively. Importantly, the mean thickness of pectinate muscles did not reflect the true wall thickness because the wall also consisted of paper-thin membranes between pectinate muscles.

Regarding the RAA apex; the anteromedial recess extended from its orifice, anteriorly, superiorly, and leftward such that it overlapped the tissues of the AV groove and the tip itself was pointing toward the RV outlet region. This was the deeper of the two recesses with a mean depth of 19.3 ± 5.2 mm measured from the origin of the SB to the tip of the anteromedial RAA recess. From an epicardial perspective the very tip of the anteromedial recess, overlies a fat pad covering the proximal portion of the right coronary artery and the AV groove (*Figure 2*). Measurements made at three different points within the anteromedial recess yielded mean inter-pectinate space wall thicknesses of 1.0 ± 0.5 mm (anterolateral segment), 0.8 ± 0.4 mm (lateral segment), and 0.9 ± 0.6 mm (posterior segment).

The posterolateral recess corresponded epicardially to the RAA summit and occupied the anterior third of the RAA upper border. Its mean depth was 10.8 ± 3.4 mm from the SB to the recess tip. When a probe was placed inside the tip of this recess, it pointed superiorly and leftward to abut the aortic root through the reflection of the visceral epicardium. The remainder of the posterolateral recess was the lateral wall of the RAA where the right phrenic nerve courses *in vivo*. The inter-pectinate wall thickness in the posterolateral recess was thinner than that in the anteromedial recess; histological examination just below the level of the SB bifurcation in the posterolateral recess showed few cardiomyocytes and sometimes a total absence of myocardium, with only epicardium–endocardium and fibrous tissue in between the very thin layers (*Figure 1*).

### Right atrial lateral wall

Right atrial lateral wall between the SVC and IVC had a mean wall thickness of 2.6 ± 0.9 mm (range 1.5–5.5 mm). This wall measurement was inclusive of epicardial fat which was highly variable in its thickness. The sinoatrial artery runs in close proximity along the epicardial aspect of the RA lateral wall.

There were no significant differences in measurements when analysed by sex. (The full dataset of measurements is available in Supplementary material online, *Table S1* and Supplementary material online, *Figure S1*.)

### Magnetic resonance imaging analysis of right atrial appendage dimensions

**Figure 3:**
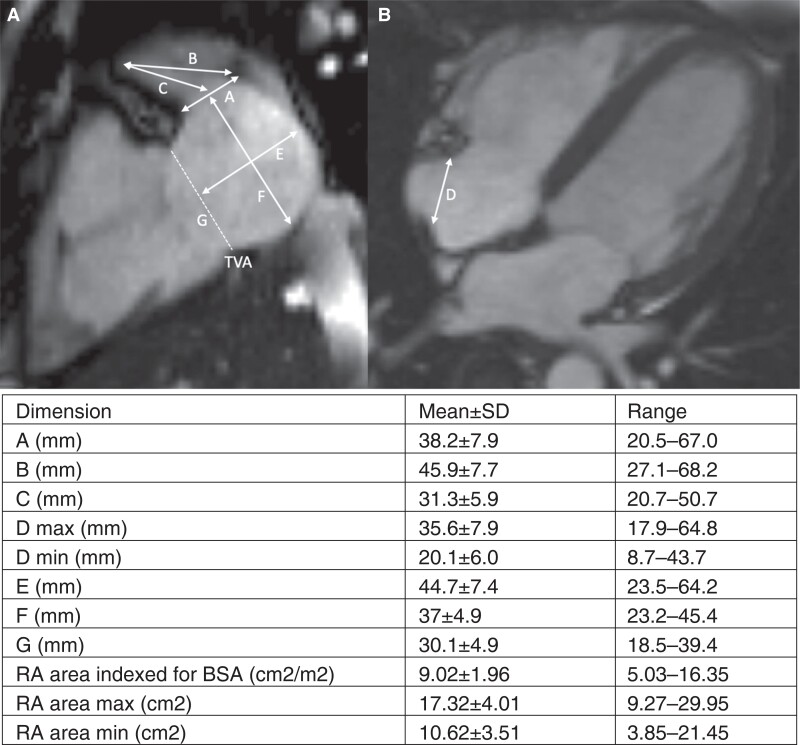
Cardiac MRI dimensions analysed. Representative MRI images demonstrating the measurements taken and the resultant mean measurement; (*A*) two-chamber view. (B) Four-chamber view. Measurements noted: A—RAA ostium, B—RAA depth to CT, C—RAA depth to mid-point of RAA ostium, D—CT to lateral TVA, E—RA diameter (2Ch view) perpendicular to TV annulus, F—RA diameter (2Ch view) perpendicular to RAA ostium, G—TVA diameter (2Ch view). BSA, body surface area; CT, crista terminalis; MRI, magnetic resonance imaging; RA, right atrium; RAA, right atrial appendage; TVA, tricuspid valve annulus.

In total, 100 patients were included in MRI portion of the study; 55% were female, the mean age was 48 ± 13 years, and body mass index (BMI) was 21 ± 1 (*Table 2*). The RAA ostium was measured in the two-chamber view as the distance between the CT prominence and the tricuspid valve (A). The two-chamber view was used to measure the depth of the RAA in terms of distance from the anteromedial tip to the CT (B) and to the mid-point of the RAA ostium (C) (*Figure 3*).

**Table 2:** Patient characteristics of the CMR cohort

Characteristics	
*n*	Total: 100
Male	45 (45%)
Age (years)	48 ± 13
Height (cm)	173 ± 9
Weight (kg)	79 ± 17
Body surface area (m^2^)	1.8 ± 0.5
Body mass index (kg/m^2^)	21 ± 1
Underlying pathology	
Non-ischaemic cardiomyopathy	33
Ischaemic heart disease	20
Brady/tachycardiac arrhythmias	15
Valvular disease	4
Hypertension	4
Healthy volunteers	3
Peri/myocarditis	3
Other	18

CMR, cardiac magnetic resonance imaging. Continuous variables presented as mean ± SD.

Mean RAA depth to the CT (B) was 45.9 ± 7.7 mm (range 27.1–68.2 mm) and the mean depth to the mid-point of the RAA ostium (C) was 31.3 ± 5.9 mm (range 20.7–50.7 mm) (*Figure 3*). The distance from the CT to the lateral tricuspid valve annulus (D) was 35.6 ± 7.9 mm (range 17.9–64.8 mm). Multivariable analysis demonstrated that the only parameter significantly related to the depth of the RAA was the body surface area (*P* = 0.04 for the distance C and *P* = 0.001 for distance B). When analysed by sex, there were no differences in dimensions (see Supplementary material online, *Table S2*).

The mean distance from the RAA apex to the SVC wall was 46 mm (range 33–55 mm) and from the RAA apex to the IVC wall was 77 mm (range 59–96 mm). Regarding the adjacent right lung, the distance from the RA anteromedial apex to the right lung (mean 9.7 mm, range 1.0–39 mm) and the RA lateral wall to the right lung (mean 3.2 mm, range 1.0–23 mm).

### Virtual implantation of an atrial leadless pacemakers

**Figure 4:**
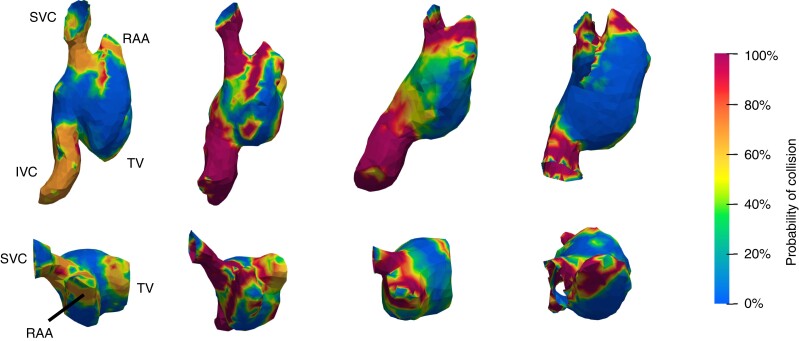
3D collision analysis: representative images from four patients of the collision analysis from 3D modelling in a right lateral (top) and superior (bottom) view demonstrating the incidence of collision of the virtual device implant throughout the cardiac cycle. A 0% incidence of contact throughout the cardiac cycle is represented in blue with a continuous scale to 100% contact throughout the cardiac cycle in dark red. (The three suggested implant locations for an atrial leadless pacemaker in this collision model are shown in supplementary material online, *Figure S2*.) IVC, inferior vena cava; RAA, right atrial appendage; SVC, superior vena cava; TV, tricuspid valve.

Regions that consistently demonstrated a low contact value (defined as contact for <20% of the cardiac cycle) included the base of the RAA, the RAA apex as well as the mid-portion of the RA lateral wall. The septum also showed a low contact value but was excluded from the analysis due to our initial practical considerations. In contrast, regions demonstrating a high contact value (defined as contact for >80% of the cardiac cycle) included the high lateral RA wall at the RA/SVC junction and the RAA body (*Figure 4*). Dynamic modelling assessment demonstrated that contact occurs just prior to, during, and just after peak atrial systole (see Supplementary material online, *Videos S1* and *S2*).

## Discussion

Atrioventricular synchrony is important for optimal cardiac function, until recently this has been the downfall of LP. The Micra AV^TM^ LP was developed to provide AV synchronization by using an inbuilt accelerometer to detect mechanical atrial activation. This device has been shown to be effective at low heart rates, but at higher heart rates may be less effective at maintaining AV synchrony.^12,13^ Furthermore, this does not provide a satisfactory solution for patients with sinus node dysfunction. There is thus an unmet need for an atrial LP that could be used as a stand-alone device or in combination with a ventricular LP.

Our study is, to our knowledge, the first study to comprehensively evaluate RA anatomy using both *ex vivo* and *in vivo* analysis in conjunction with dynamic 3D modelling with regard to an atrial LP implant. The key findings from this multimodality assessment include the identification of the RAA base, RAA apex (with preference to the anteromedial recess), and RA lateral wall as favourable locations based on a combination of consistent wall thickness, accessibility with existing delivery tools, and minimized mechanical interactions with other RA structures or the tricuspid valve apparatus (*Table 3*). In contrast, the RA septum, proximal CT, or RAA body were less favourable due to theoretically increased risks for perforation, phrenic capture, or mechanical interference. Notably, we did not identify important differences between men and women, suggesting that a single delivery system and LP design may be suitable for all patients.

**Table 3:** Summary of potential RA LPM implant locations

Location	Wall thickness	Device contact (per cardiac cycle) (%)	Potential perforation risk	Potential dislodgement risk	Other potential risks	Potential advantages
RAA base	4.2 ± 1.4mm	<20	Low	Low		Easy implant (and retrieval) approach
RAA apex (anteromedial recess)	1.0 ± 0.5mm	<20	Low–moderate	Low–moderate	Poor device orientation for inter-device communication	Will accommodate whole device within RAA (low dislodgement risk)
RA lateral wall	2.6 ± 0.9 mm	<20	Low	Moderate	Risk of PNS	
RAA body	Paper thin between trabeculations	>80	High	Moderate	Risk of RAA perforation with rear of device	
Superior CT	4.2 ± 1.4 mm	>80	Low	Moderate	Variable location	Easy implant (and retrieval) approach
					Absent in 12%	
					Difficult to target (small region)	
					High risk of PNS	
Atrial septum	Not studied	<20	Low	High	Inadvertent LA placement (PFO/ASD)	

ASD, atrial septal defect; CT, crista terminalis; LPM, leadless pacemaker; PFO, patent foramen ovale; PNS, phrenic nerve stimulation; RA, right atrium; RAA, right atrial appendage.

Potential adverse events to consider with an atrial LP are strongly influenced by local anatomy and include cardiac perforation, right-sided pneumothorax, aortic injury, coronary sinus obstruction, and interaction with, or obstruction of, the tricuspid valve. The risk of each of these potential complications can be minimized by a deep understanding of the atrial anatomy that we present. The importance of detailed consideration of the implant location is highlighted by the initial high rate of cardiac perforation with the Micra (Medtronic, Minneapolis) RV LP prompting a change in practice with the planned implant location moving from the RV apex to the septum.^5,6,14–16^ Experience from leadless pacemaker (LPM) implants in the RV should also be considered when evaluating the future patient cohort for atrial LPMs. For example, the patient factors identified by Piccini *et al*.^16^ for pericardial effusion development [age >85 years, chronic obstructive pulmonary disease (COPD), BMI <20 kg/m^2^] will likely also apply to atrial LPMs. However, this small increased procedural risk needs to be balanced against the reduction in long-term complications in high-risk patients (end-stage renal disease, dialysis, tricuspid valve disease, concurrent malignancy, and COPD) that leadless pacing offers.^14^

The additional device-related complication is that of device dislodgement. From our data, it is possible that the RAA apex, being 19.3 ± 5.2 mm in depth may provide increased stability as almost the entire device will fit within the RAA itself. In both the RAA base and RA lateral wall positions, the body of the device will be located within the main body of the RA. However, to assess this further, an *in vivo* study would be required to confirm if this theory translates into clinical reality.

Conceptually, there are four possible implant locations for a 32 mm long RA LP with a 1.6 mm fixed helix; the superior aspect of the CT at its bifurcation with the SB (at the RA/SVC junction), the lateral RA wall, RAA apex, and RAA base. Our analysis refines these putative implantation sites with important local observations. The superior CT may be attractive due to its significant wall thickness (4.2 mm), yet anatomical variation between subjects means that in some cases the SB is attached very posteriorly, or is absent making difficult to clearly distinguish the thicker region. Furthermore, the CT/SB bifurcation was absent in 12% of our specimens, and the lack of a distinct bifurcation inherently reduces the target area where the wall thickness is greatest. It is also very close to the upper crest of the RAA and thus is directly adjacent to the ascending aorta raising the possibility of injury to, or perforation of, the aortic root during implantation. The right phrenic nerve may also pass in the vicinity, and it has been demonstrated in electrophysiology studies that during pace-mapping one is able to capture and stimulate the right phrenic nerve in 100% of patients from the upper RA/SVC area.^17^ Thus, placement here could result in inadvertent phrenic nerve stimulation or mechanical damage due to helix perforation from an LP placed in this location. In addition to the right phrenic nerve, other epicardial structures including the sinus node and the sinoatrial nodal artery are in close proximity to this location, injury to the latter may result in atrial infarction and non-viability of the same region in which the LP is placed. Finally, 3D collision modelling of the superior CT region suggests a high incidence of interaction with other RA walls which may increase the risk of late cardiac perforation.

An alternative implant location is the base of the lateral RAA, the pectinate muscles, and atrial wall are of acceptable wall thickness of 2.7 ± 1.6 mm (range 1–8.5 mm) which would, in most cases, accept the 1.63 mm fixed helix. Furthermore, the collision analysis is favourable in this location. An implant at the RAA base would be quite direct from an inferior approach (the sheath deflection would be minimal), and this technical advantage is likely to pertain additional safety advantages during the implant procedure itself as aggressive sheath flexion in the RA/RAA will increase the risk of mechanical perforation.

Regarding the RAA apex, the posterolateral recess of the RAA was thinner and with relatively widely spaced pectinate muscles, increasing the potential risk of perforation during implantation. Its crest lies adjacent to the aortic root, and active fixation leads placed here have been demonstrated to result in aortic perforation.^18^ The right phrenic nerve courses down the lateral wall of the posterolateral RAA and may potentially be directly injured during implantation or inadvertently captured during pacing from this location as well. The anteromedial recess of the RAA has the advantage of tightly packed pectinated muscles and a thicker wall decreasing the risk of perforation during implantation, furthermore the consequences of a perforation of the helix at the tip of the anteromedial recess are minimized by the presence of epicardial fat which may act to protect the nearby proximal right coronary artery and prevent pericardial perforation.

The mid-region of the RAA has a wider spacing of the pectinate muscles with mean wall thickness of the pectinate muscles less than the 1.6 mm threshold of the considered helix. Furthermore, the inter-pectinate space had a paper-thin wall which would be highly susceptible to perforation. Finally, interaction of the 32 mm long device in our 3D modelling in this location was high confirming this location to be sub-optimal as an implant location.

The depth of the anteromedial recess was 19.3 ± 5.2 mm (*ex vivo*) or 31.3 ± 5.9 mm (*in vivo*) in our study suggesting that a minimum of 2/3 of an LP would be contained within this structure. Conceivably, in this position device movement would be reduced as its entire length would be resting on the pectinate muscles with the added advantage that it would not push on the paper-thin tissue between the PM. However, this ‘protected’ site may have slower blood flow velocities which would potentially pre-dispose to device-related thrombus. In the cases of collision modelling, there was a very low collision likelihood when the LP was placed in the RAA apex. However, body surface area (BSA) predicted the depth of the RAA, thus patients with a smaller BSA may be at higher risk of the LP protruding into the vestibule of the RA and thus interacting with other intra-cardiac structures.

The RA lateral wall had a similar wall thickness to the RAA apex, but these measurements include the epicardial fat in this region thus it is conceivable that implantation in this location may still perforate the epicardial layer; the degree of clinical consequence of this micro-perforation is uncertain. Similar to the CT/SB bifurcation, the lateral wall location also has concerns regarding phrenic nerve stimulation, though endocardial capture of the phrenic is less consistent at the mid-atrial level compared to the superior RA/SVC junction. The RA lateral wall has the advantage of being easily accessible from an inferior approach (though not as direct as the RAA base) and a very low incidence of collision in virtual implantations in this area suggesting the device would not be subject to mechanical trauma.

LPM dislodgement is a potential concern, but there have been no reports of external cardioversion resulting in ventricular leadless pacemaker dislodgement (which have a similar fixation mechanism) and no cases of atrial LPM dislodgements in the first 300 implants and as such we believe the risk to be low.^19^

Though we did not evaluate the electrophysiological properties of the different implant locations, the inter-atrial and intra-atrial conduction is affected by atrial pacing location. Inter-atrial conduction is rapid through the use of Bachmann’s bundle and the coronary sinus musculature, conversely RAA pacing is associated with inter-atrial conduction delay which predisposes to AF and may adversely affect atrial fibrillation timing.^20,21^ Even in the case of traditional lead-driven pacing, the optimal atrial pacing site regarding atrial conduction has not been established. The atrial sensing and pacing thresholds may be affected by the wall thickness and be a consideration regarding implant location choice, with LPs the smaller battery this aspect may be important considering device longevity.

Given that the optimal pacing site in the RA has not been established traditional atrial lead placement in the RAA apex and RA lateral wall is most common, the RAA base and CT bifurcation are infrequently targeted. This is based on the ease and success of lead implant from a superior approach. An atrial LP is likely to be predominantly implanted from an inferior approach due to sheath size and thus the ease of implanting (and potentially extraction) in any one location will not be the same.

Given all the factors discussed the RAA base may be the best compromise of all potential implant locations. However, the lateral RA wall and anteromedial recess of the RAA apex may also be considered. The communication between the two leadless devices is influenced by the orientation of the devices with regard to each other, this aspect may favour placement on the RA lateral wall of the atrial LP. Furthermore, the placement and orientation of the RV LP should be considered when anticipating inter-device communication.

Knops *et al.* have recently published the early outcome data from the first in man trial of an atrial LPM with an impressive success rate of 98.3% over 300 cases. The requirement of intra-procedural atrial LPM re-positioning was approximately double compared to the ventricular LPM (24.2% vs. 10.4%). Similarly, atrial LPM dislodgement was more common (3.3% vs. 0.3%), and pericardial effusions occurred in 0.7% of patients. The increased complication rate with the atrial LPM device highlights the importance of a deep anatomical understanding of the RA specifically relating to LPM implantation. The authors observed a numerically higher rate of dislodgements from the RAA tip location (4.4% vs. 0.9%). Based on our data, the increased rate of dislodgement may be due to insufficient RA wall thickness to anchor the device (only 1.0 ± 0.5 mm). Our recommendation based on anatomical and modelling data confirms and expands on the recommendation of the RAA base as the favoured implant location.^19^

Potential clinical implications of our findings include anticipated use of pre-procedure imaging with MRI or CT to guide decision-making regarding individual device placement to identify the presence of clear SB boundaries, RAA size, and wall thickness at potential implant locations. Intra-cardiac echocardiography in real time during implantation may also be useful, particularly early in individual operators’ learning curves or in further pre-market series in which implantation technique and the delivery systems themselves may undergo important refinement. Imaging-guided implantation of atrial LPs as routine practice would limit the implanting institutions significantly; once the early experience of implants has been established, it is likely that pre-implant imaging, beyond echocardiography, is unlikely to be required. During the implant at the RAA base, we would advocate for precise confirmation of final positioning before deployment by means of two orthogonal views (left anterior oblique view to confirm the lateral approach during the initial delivery, right anterior oblique projection to confirm that it is anterior enough) and the use of contrast dye injection during the intervention may permit a very high anatomical definition of the RAA morphology relevant to the implantation of the LP (see Supplementary material online, *Figure S3* and *Videos S3* and *S4*). As experience grows with this technology implantation without specialist imaging may become more routine allowing the expansion of atrial LPs to centres and countries without access to such imaging.

### Limitations

The studied specimens were fixed in formalin which results in measurements that are smaller than in living tissue due to tissue contraction. However, it has been demonstrated that 10% formalin solution is the best fixative for cardiac morphometric purposes because this solution causes the smallest changes in tissue dimensions, provided that measurements are obtained at least after 1 week of preservation; all our samples were in fixative solution more than 1 week before the study.^22^ The specimens we studied did not include those with significant structural heart disease, the mean age of the MRI cohort was 48 years and that of the modelling cohort was 33 years and so application of our results to the wider population should be performed with caution. We have modelled the physical aspects of an atrial LP but have not included an assessment of atrial blood flow dynamics. Though there is a potential for atrial blood flow dynamics to influence the position and movement of an atrial LP, the low velocity and pressure of the RA cavity mean this factor is likely to have only a very minor, if any, clinical effect. We did not take into consideration the potential impact that the relative orientations of the RV and RA LPs may have on inter-device communication.

## Conclusions

Based on anatomical review and 3D modelling, we conclude that the theoretical optimal location for an atrial LP implant is either the base of the RAA, the anteromedial RAA apex, or the RA lateral wall (*Graphical Abstract*). These locations give the most attractive safety profile and physical compatibility with the RA based on a virtual implant (see Supplementary material online, *Figure S2*). The mid-RAA, RA/SVC junction, and septum appear to be sub-optimal fixation locations. The base of the RAA may provide the best compromise of all the implant considerations. Future studies are indicated to evaluate the *in vivo* feasibility and safety of atrial LP technology.

## Supplementary Material

euad235_Supplementary_Data

## Data Availability

The data that support these findings are available from the corresponding author (TW) upon reasonable request.

## Appendix

For the MRI component of this study, the inclusion criteria were age >18 years old, sinus rhythm during the cardiac MRI, and absence of severe structural heart disease. Exclusion criteria included clinical heart of failure, congenital heart disease, severe valve disease, moderate/severe reduction of left ventricle ejection fraction, any impairment of the RV systolic function, dilated cardiac chambers, pericardial effusion, pericardial constriction, or lack of pericardium.
