# Supplementary material for: Anatomic, histologic, and mechanical features of the right atrium: implications for leadless atrial pacemaker implantation
Source: Europace. 2023 Jul 31;25(9):euad235. doi: 10.1093/europace/euad235 (PMC10473833; doi:10.1093/europace/euad235)
Supplement: euad235_Supplementary_Data [file euad235_supplementary_data.zip › Supplementary Material.docx]

Supplementary Table 1 Anatomical measurements of the right atrium:

| **Figure reference** | **Anatomical location** | **Distance (mm)** |
| --- | --- | --- |
| 1 | TV annulus (diameter from the membranous septum) | 31.1±5.7 |
| 2 | TV annulus (diameter from level of coronary sinus orifice) | 28.4±6.7 |
| 3 | Depth of the posterolateral part of the RAA tip (from the apex to the CT-SB bifurcation) | 10.8±3.4 |
| 4 | Depth of the anteromedial part of the RAA tip (from the apex to the CT-SB bifurcation) | 19.3±5.2 |
| 5 | Width of the proximal site of the SB | 5.0±1.3 |
| 6 | Length of the SB from the CT-SB bifurcation to the point where the SB branches become indistinguishable, mingling with surrounding pectinate muscles, or branch, or insert into the vestibule | 9.8±3.6 |
| 7 | The internal distance between the apex of the anteromedial part of the RAA tip and the Eustachian valve (or junction between IVC and RA) | 58.1±8.3 |
| 8 | The external length of the sulcus terminalis | 36.4±6.5 |
| 9 | Width of the of proximal site of the CT (SB/CT bifurcation) | 6.0±1.5 |
| 10 | Width of the CT 10mm from the proximal site | 5.6±1.4 |
| 11 | External length from RAA apex to IVC/RA junction | 59.8±7.9 |
| 12 | External length from RAA apex to SVC/RA junction | 29.6±4.7 |
| 13 | Distance between the Oval Fossa and the apex of the anteromedial part of the RAA tip | 50.0±7.9 |
| 14 | Distance between the TV annulus and the apex of the anteromedial part of the RAA tip | 37.0±6.3 |
| 15 | Diameter of the orifice of the anteromedial part of the RAA tip | 13.3±2.9 |
| 16 | Diameter of the orifice of the anteromedial part of the RAA tip (orthogonal to above) | 12.2±2.9 |
|  |  |  |
|  | **Anatomic point** | **Wall thickness** |
| 17 | Thickness of the of proximal site of the CT (SB/CT bifurcation) | 4.9±1.9 |
| 18 | Thickness of the CT 10mm from the proximal site | 4.2±1.4 |
| 19 | Thickness of the CT at the level of inferior vena cava junctions with RA | 2.5±0.9 |
| 20 | Thickness at the of proximal site of the SB | 3.8±1.1 |
| 21 | Thickness of the inter-caval space | 2.7±1.6 |
| 22 | Thickness of the wall inside the RAA anteromedial tip just near the SB | 1.0±0.5 |
| 23 | Thickness of the wall inside the RAA anteromedial tip at the very apex | 0.8±0.4 |
| 24 | Thickness of the wall inside the RAA posterolateral | 0.7±0.4 |
| 25 | Thickness of the wall inside the RAA anteromedial tip near the medial side of the orifice | 0.9±0.6 |
| 26 | Thickness of the wall (pectinate muscles) in the anterolateral segment | 1.3±0.4 |
| 27 | Thickness of the wall (pectinate muscles) in the lateral segment | 1.2±0.3 |
| 28 | Thickness of the wall (pectinate muscles) in the posterior segment | 1.3±0.4 |

Supplementary Table 2: MRI patient characteristics and dimensions analysed by sex

|  | Male (n=55) | Female (n=45) | p-value |
| --- | --- | --- | --- |
| Age (years) | 47±16 | 50±15 | 0.438 |
| Height (cm) | 178±8 | 166±6 | <0.001 |
| Weight (kg) | 86±16 | 71±16 | <0.001 |
| BSA (m^2^) | 1.96±0.4 | 1.69±0.4 | 0.002 |
| BMI (kg/m^2^) | 20.1±1.2 | 22.2±4.3 | <0.001 |
| A (mm) | 38.5±7.7 | 37.7±8.1 | 0.62 |
| B (mm) | 47.2±8.3 | 44.2±6.6 | 0.05 |
| C (mm) | 31.9±6.2 | 30.5±5.3 | 0.22 |
| D max (mm) | 37±7.4 | 33.8±8.2 | 0.04 |
| D min (mm) | 20.8±6.7 | 19.3±5 | 0.231 |
| E (mm) | 45±7.2 | 44.2±7.6 | 0.50 |
| F (mm) | 36.4±5.4 | 37.7±4.3 | 0.19 |
| G (mm) | 29.9±5.4 | 30.4±4.3 | 0.63 |
| RA area indexed for BSA (cm^2^/m^2^) | 9.1±1.7 | 8.9±2.3 | 0.779 |
| RA area max (cm^2^) | 18.3±3.8 | 15.9±3.9 | 0.003 |
| RA area min (cm^2^) | 11.8±3.8 | 9.2±2.5 | <0.001 |

Supplementary Table 3: Collision modelling patient characteristics

| Case | Sex | Age |
| --- | --- | --- |
| 1 | F | 31 |
| 2 | M | 29 |
| 3 | M | 33 |
| 4 | F | 28 |
| 5 | M | 35 |
| 6 | F | 35 |
| 7 | M | 37 |
| 8 | F | 36 |
| 9 | M | 34 |
| 10 | F | 30 |

Supplementary Figure 1: Right atrial anatomical measurement locations:


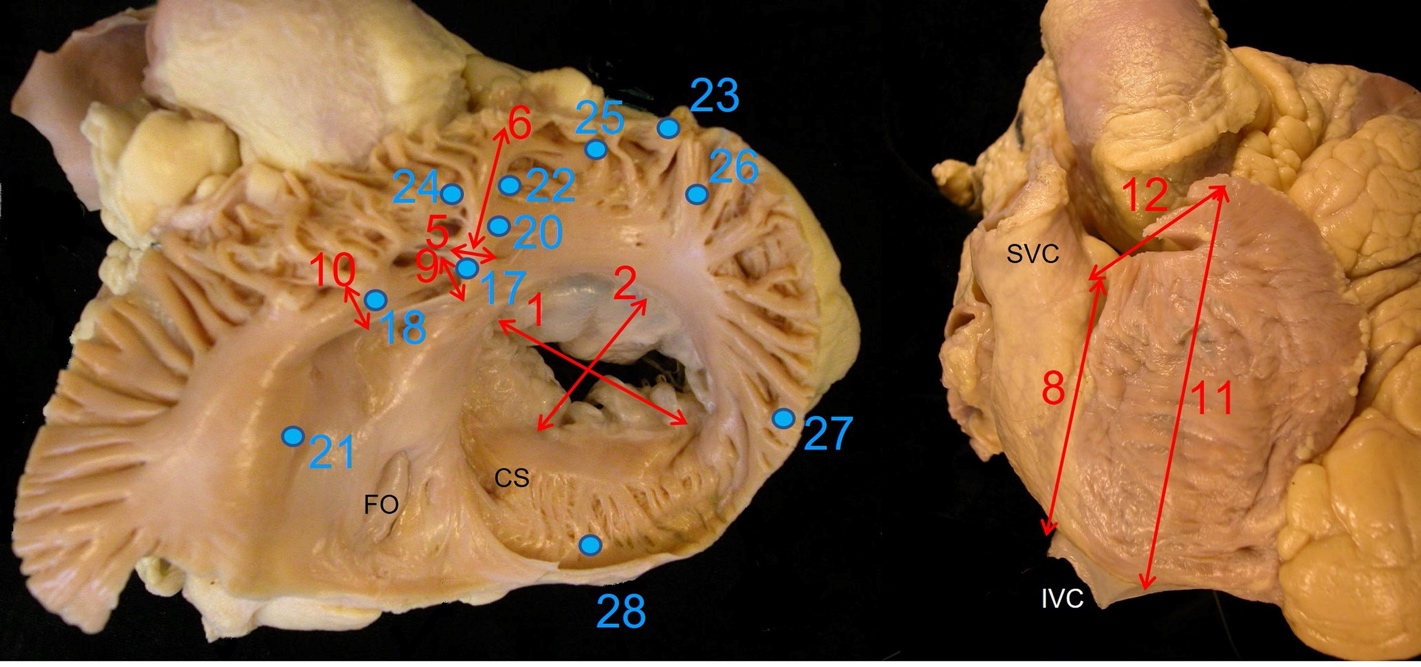


Internal and external views of right atrium showing most of the sites of measurements listed on Supplementary Table 1. CS – coronary sinus, FO – *fossa ovale*, IVC – *inferior vena cava*, SVC – *superior vena cava.*

Supplementary Figure 2: Three suggested implant locations on the collision model.


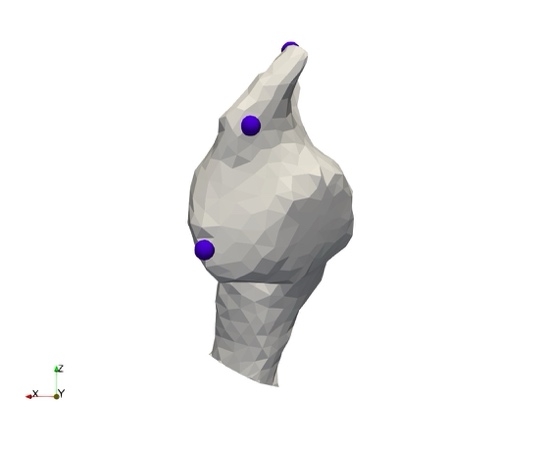

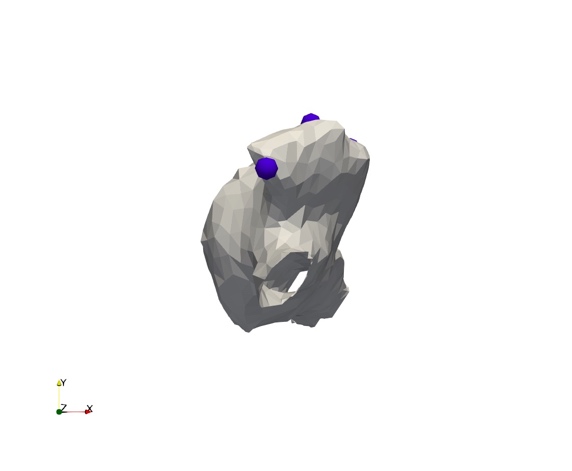


The purple dots represent the three suggested locations for an atrial LPM implant (base of the RAA, the anteromedial RAA apex or the right atrial lateral wall)

Supplementary Figure 3: Three suggested implant locations on fluoroscopy


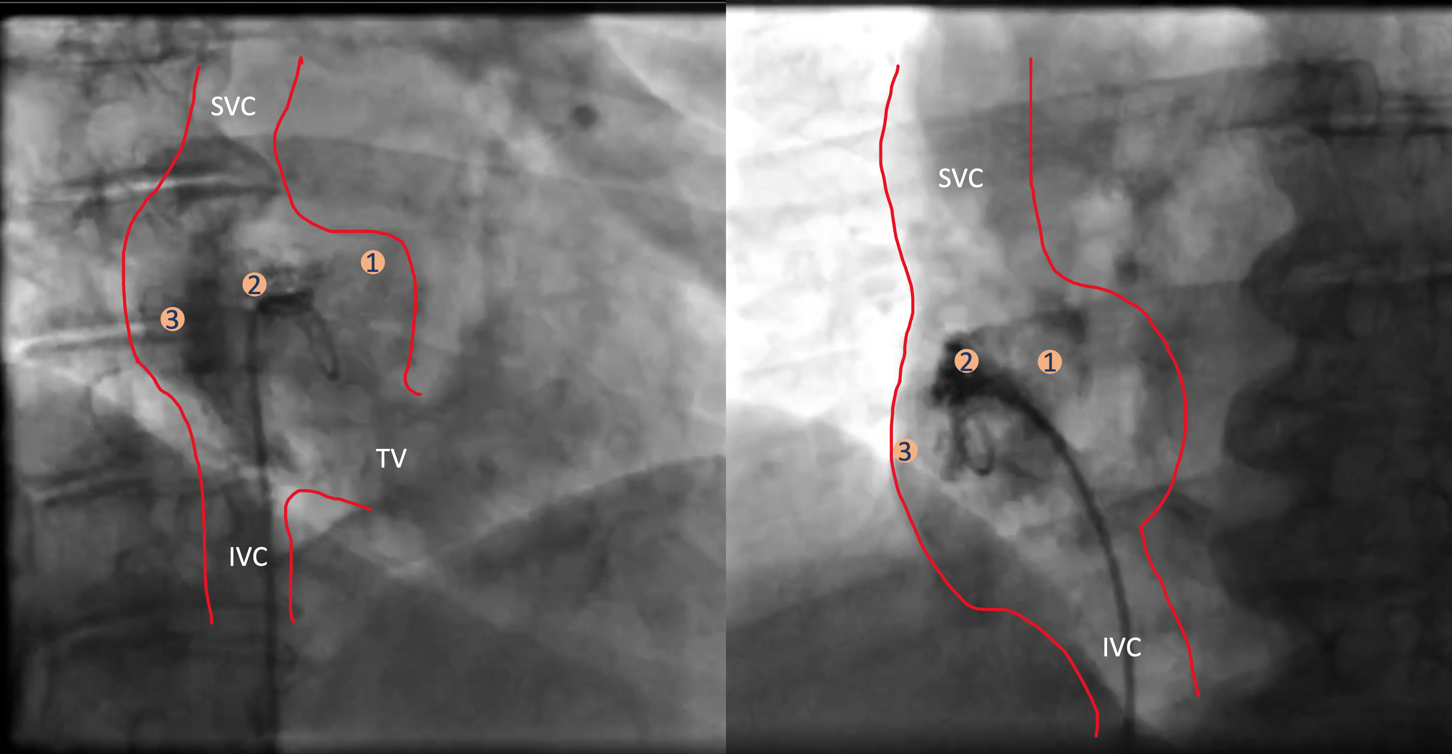


RAO (left) and LAO (right) fluoroscopic images with the three potential RA LPM implant sites highlighted. (1) Anteromedial RAA apex (2) base of the RAA (3) Right atrial lateral wall.

Supplementary Video 1: Right lateral view of the dynamic collision analysis. Binary collision analysis performed at each vertex of the triangulated RA mesh at each stage of the cardiac cycle (red representing collision and green representing no collision). Regions of minimal collision throughout the cardiac cycle include the RAA apex, RAA base and the RA lateral wall. RA – Right Atrium

Supplementary Video 2: Superior view of the dynamic collision analysis. Binary collision analysis performed at each vertex of the triangulated RA mesh at each stage of the cardiac cycle (red representing collision and green representing no collision). Regions of minimal collision throughout the cardiac cycle include the RAA apex, RAA base and the RA lateral wall RA – Right Atrium

Supplementary Video 3: RAO fluoroscopic contrast injection to outline the RAA anatomy. The trabeculated nature of the RAA can be appreciated. In this RAO projection the apex of the RAA can be confirmed as the most anterior and portion of the RAA, the base of the RAA as the most posterior aspect and the region of the RA lateral wall can be seen posterior to the RAA and tricuspid valve plane. RAA – Right Atrial Appendage, RAO – Right Anterior Oblique.

Supplementary Video 4: LAO fluoroscopic contrast injection to outline the RAA anatomy. Here the lateral wall of the RA can also be appreciated as the right edge of the cardiac silhouette. RAA – Right Atrial Appendage, LAO – Left Anterior Oblique.
